# Impact and acceptability of self-consent procedures for the school-based human papillomavirus vaccine: a mixed-methods study protocol

**DOI:** 10.1136/bmjopen-2017-021321

**Published:** 2018-03-03

**Authors:** Suzanne Audrey, Harriet Batista Ferrer, Joanne Ferrie, Karen Evans, Michael Bell, Julie Yates, Marion Roderick, John MacLeod, Matthew Hickman

**Affiliations:** 1 Population Health Sciences, Bristol Medical School, University of Bristol, Bristol, UK; 2 Screening and Immunisations South West, Public Health England, Bristol, UK; 3 Head of School Nursing (South Gloucestershire) and Specialist Nursing Services, South Gloucestershire, UK; 4 Bristol Biomedical Research Centre and NIHR CLAHRC West, Bristol, UK; 5 Department of Paediatric Immunology, Bristol Children’s Hospital, Bristol, UK

**Keywords:** health policy, sexual medicine

## Abstract

**Introduction:**

The human papillomavirus (HPV) vaccine, administered in early adolescence, can substantially reduce cervical cancer incidence and mortality. However, lack of written parental consent is a key reason why some young women do not receive the vaccine. The national legal framework allows girls to be vaccinated without parental consent provided they are deemed Gillick competent, but there is some reticence about vaccinating without written parental consent. Self-consent procedures are being implemented in Bristol and South Gloucestershire. This study will examine the implementation, acceptability and impact of these new procedures.

**Methods and analysis:**

Statistical analyses of routine data from Public Health England and the Child Health Information System will test if there has been an increase in HPV vaccination uptake in two ways: (a) Is there an increase when comparing before and after the change in our intervention sites? and (b) Does the percentage change in our intervention sites differ from comparison sites (similar to our intervention sites in terms of initial HPV uptake, ethnicity and deprivation levels) in England where no such intervention took place and how? For the process evaluation, we will develop a logic model and use questionnaires, observations and audio-recorded interviews with young women, school nurses, school staff and parents to examine the context, implementation of self-consent and response to the new procedures.

**Ethics and dissemination:**

The University of Bristol Faculty of Health Sciences Research Ethics Committee and the National Health Service Health Research Authority provided approvals for the study. We will produce a report with recommendations about self-consent procedures in conjunction with key stakeholders. At least two papers will be written for publication in peer-reviewed journals and for conference presentations. A summary of results will be shared with participating immunisation nurses, school staff, young people and parents as requested.

**Trial registration number:**

ISRCTN49086105; Pre-results.

**Strengths and limitations of this study**

- The study addresses the lack of evidence about the impact and acceptability of self-consent procedures for the school-based human papillomavirus vaccination programme.
- The study will examine new self-consent procedures as they are implemented in routine practice.
- Although not a trial, the proposed statistical analyses of routine data will provide evidence of the impact of self-consent procedures on vaccine uptake.
- The process evaluation will identify barriers and enablers to self-consent for adolescent vaccinations.
- Findings from this mixed-methods study will inform recommendations for future practice, and may be relevant to other adolescent vaccination programmes.

## Introduction

### Inequalities in uptake of the human papillomavirus vaccine

High coverage of human papillomavirus (HPV) vaccination programmes in early adolescence can substantially reduce cervical cancer incidence and mortality.^1 2^ In the UK, the HPV immunisation programme, predominantly delivered through schools, is achieving overall high coverage, and estimates suggest it will save 400 women each year from developing cervical cancer.^3^ However, research suggests lower socioeconomic status (SES) and some ethnic groups are associated with lower uptake of HPV vaccination.^4 5^ The incidence of cervical cancer has been found to be higher in lower socioeconomic groups^5^ and these women are also less likely to access screening programmes than higher socioeconomic groups contributing to a higher mortality rate.^6^ Inability to access vaccination adds a further layer of health inequality. Furthermore, students in special schools or pupil referral units are much less likely to receive the vaccine^6^ and may require additional efforts by healthcare professionals to ensure they are vaccinated.^7^

Because of the preferred age for HPV vaccination (12/13 years), written parental consent tends to be sought. However, previous research examining facilitators and barriers to uptake of the HPV vaccine has shown that a lack of written parental consent is a key reason why some young women do not receive the vaccine.^8 9^ Furthermore, it is a barrier with potential to reinforce health inequalities since lack of written parental consent may also be related to lower SES and some ethnic groups.^8 9^

### Self-consent for HPV vaccination

In the UK, the legal framework allows girls to be vaccinated without parental consent provided they are deemed Gillick competent.^10^ However, WHO has acknowledged difficulties over consent for HPV vaccination because of the age of the target group, and suggests, at the very least, parents should be informed of the planned vaccination to provide an opportunity for the child to ‘opt out’ of the procedure.^11^ In England, National Health Service information about HPV vaccination states: “Although, as a parent, you’re asked to sign a consent form, it is up to your daughter whether she has the vaccine or not”^12^ and in Public Health England (PHE) guidance for healthcare professionals there is a lack of a clear directive about whether parental consent is necessary.^13^

This lack of clarity has implications for the vaccination process. Parental consent may be missing because of problems in returning paperwork, or because a parent is unwilling to allow their daughter to be vaccinated. In both cases, there may be young women who wish to receive the vaccination. However, some immunisation nurses and school staff appear reluctant to allow girls to make their own decisions about HPV vaccination because of concerns about generating antagonism between parents and the school or healthcare providers.^8^

The issue of vaccination without written parental consent needs clarification, and guidance about self-consent procedures is required. However, there is a paucity of peer-reviewed published research on the topic of self-consent for adolescent vaccinations. A study undertaken in the USA concluded that the inability of minors to consent for vaccines is a likely barrier to vaccination, and that interventions to increase adolescent vaccination should consider strategies that increase the ability of unaccompanied minors, particularly older minors, to receive vaccines within the context of legal, ethical and professional guidelines.^14^ Also in relation to the USA, Dempsey and Zimet suggest the debate over whether adolescents should be legally allowed to self-consent to vaccination is unresolved and could have a substantial impact on vaccination rates.^15^

In her doctoral thesis, Batista-Ferrer^16^ examined consent for the HPV vaccine in schools in Bristol, UK. Reasons recorded for why eligible girls did not receive the first dose of the HPV vaccine course revealed that lack of a signed parental consent form was the main reason (45.9%), while active refusal by parents occurred much less frequently (11.9%). Nevertheless, research indicates that immunisation nurses and school staff were unwilling to be held accountable if young women presented for HPV vaccination without parental consent.^7 16^

PHE data for 2014/2015 show some areas in the south-west of England with low uptake of HPV vaccination. For example, Bristol was ranked 112th of 119 English local authorities (LAs) (excluding London) and South Gloucestershire was 106th. Because of concerns about low uptake rates, staff at PHE (South West) developed a ‘South West Template Pathway on Self Consent for School Aged Immunisations’. The aim is to support provider organisations in implementing a self-consent process to support young people to easily access vaccines, support immunisers to feel confident about self-consent and to improve the uptake of immunisations.

The current research will focus on the practicality and acceptability of implementing the new self-consent procedures, and the potential impact on overall uptake and health inequalities. This will involve a systematic review of evidence relating to consent procedures for adolescent vaccinations, a process evaluation examining new self-consent procedures in two LAs in the south-west of England and an assessment of the impact of self-consent on overall uptake levels and in relation to SES, ethnicity and type of school.

## Methods and analysis

The full study comprises three key elements: a mixed-methods systematic review of the literature relating to adolescent self-consent for vaccines; process evaluation establishing a logic model for self-consent and examining the context, delivery and response to self-consent procedures for the HPV vaccination programme and statistical analyses of routinely collected data relating to HPV vaccination uptake. A protocol for the systematic review will be published elsewhere. Here, we focus on the statistical analyses of routine data and the process evaluation in Bristol and South Gloucestershire LAs.

### The new self-consent procedures

Information about the HPV vaccine, together with forms requesting parental consent, is distributed to young women at school to take home to their parents or carers. Previously, young women who had not returned a written parental consent form were not administered the vaccine at school. Under the new arrangements, information and parental consent forms are still provided by the school for the young women to take home to their parents or carers. Those who return a completed parental consent form agreeing for vaccination to take place are included in the school-based vaccination session, but in addition those who do not have a signed parental consent are also asked to attend the session where an attempt is made by the immunisation nurse to gain verbal parental consent over the telephone. Where this is achieved, the young women are offered the HPV vaccine. If the parent cannot be contacted, the immunisation nurse assesses the young woman’s competence using a checklist and records this in a form. If the young woman is deemed competent and wants to receive the vaccine, she is asked to sign the form. If she is not deemed competent, or there is reason to believe that it would cause problems at home if she received the vaccine without parental consent, the young woman is not given the vaccine. These young women are given information about alternative options to receive the vaccination, such as through a community-based clinic run by the immunisation team: a letter with information about dates is given to the young women at the school-based session.

### Statistical analysis of routine data

Statistical analyses will examine whether the intervention (implementation of self-consent) is associated with an increase uptake overall and whether it has the potential to reduce health inequalities.

Routine PHE surveillance reports HPV uptake by geographical area but does not provide data on uptake by other factors such as ethnic group, social position or type of school. We will examine HPV uptake by these factors by extracting information from the Child Health Information System (CHIS).^17^ Following on from our previous work,^5^ we will examine data in relation to the programme years 2014/2015 until the 2018/2019 to compare uptake since the two-dose HPV vaccination programme has been implemented. The data will be anonymised and transferred in an encrypted format from the host organisation to the University of Bristol where it will be securely stored according to the Data Protection (Amendment) Act 2003 and University of Bristol requirements.

The following variables will be available from PHE through extracting anonymous aggregate information from routine immunisation systems and CHIS: number of young people offered HPV vaccination; HPV uptake by source (school, primary care, other); consent (parental written, parental verbal, young women’s self-consent); ethnic group; index of multiple deprivation^18^; childhood vaccinations; school year; year of birth; school; local government area. These data can then be used in analyses of HPV uptake. Measures of effect will be expressed as ORs and risk differences (ie, percentage of HPV uptake before intervention minus percentage of HPV uptake during intervention).

We will test whether there has been an increase in the uptake of the HPV vaccination programme before and after the intervention in terms of risk difference (difference in two proportions and tests of null hypothesis that there has been no change in uptake). Initiation of HPV vaccination uptake is 84.5% in Bristol and 86.4% in South Gloucester. Each year, approximately 1900 and 1300 (overall 3200) are invited to participate.^19^ Therefore, there will be an 80% power to detect an increase in HPV vaccine uptake to the average in England (89.4%) and over 90% power to detect an increase to 95%. In addition, we will examine whether uptake among young people from less affluent areas, minority ethnic groups and in alternative educational settings has increased and whether there has been any unintended increase or reduction in health inequalities in relation to HPV uptake. We also will examine uptake for specific schools exposed to the intervention and assess whether there is an association between the intensity of implementation of self-consent procedures (as examined in the process evaluation) and HPV vaccine uptake.

The observational data will test whether there has been an increase in HPV vaccination uptake in two ways. First, is there an increase before and after the change in our intervention sites? Second, is there evidence that the percentage change in our intervention sites is different from other sites in England where no such intervention took place? In the second set of analyses, we will seek to select comparison sites that are similar to our intervention sites in terms of ethnic diversity and social position.

### Process evaluation

The process evaluation will be conducted in line with the Medical Research Council guidance on process evaluation of complex interventions.^20^ An initial logic model will be developed, in consultation with health professionals, to show the hypothesised links between planned activities and inputs (self-consent procedures) and the sequence of short-term and medium-term outcomes (HPV vaccine implementation and uptake) that lead to desired longer-term outcomes (increased uptake, reduction in health inequalities). The initial logic model will be refined through experience and discussion as the study progresses, leading to an agreed logic model at the end of the study.

Context will be considered in relation to the socioecological model and will include examination of policy, organisational, interpersonal (family and peers) and intrapersonal factors. Questionnaires will be sent to a key contact in all secondary schools and relevant immunisation nurses in the two LAs. These will include questions about policies and procedures for HPV vaccination within schools. In addition, the researchers will compile fieldnotes when conducting observations and interviews in a sample of schools. Context will also be explored through interviews with a sample of immunisation nurses, school staff, young people and parents.

Similarly, the implementation and response to the new consent procedures will be examined through: questionnaires sent to a key contact in all secondary schools (including alternative educational settings) (n=58) and relevant immunisation nurses (n=5) in the two LAs; audio-recorded interviews (face to face or telephone) with relevant immunisation nurses, and more detailed qualitative research in eight schools (two mainstream and two alternative educational settings in each LA) purposively sampled in relation to type of school, HPV uptake, free school meal entitlement and percentage of students from minority ethnic groups. In these schools, more in-depth research will entail: audio-recorded focus groups or interviews (as preferred by participants) with approximately six young women; audio-recorded interviews with key school staff with responsibility for organising HPV vaccination sessions (one per school); observations of the process and setting for HPV vaccination and audio-recorded interviews or focus groups (as preferred by participants) with a purposive sample of approximately six parents per school.

Questionnaire responses will be reported descriptively, showing frequencies and percentages, and further illuminated by relevant free-text responses. All focus group and interview recordings will be transcribed verbatim and any potentially identifying information removed. Familiarisation with the data will involve two researchers reading and discussing the transcripts to compare and begin to code and categorise the data. Thematic analysis will be undertaken, assisted by the Framework approach to data management.^21^ Primary charts will be created using sections of the text relating to the context, views and experiences of self-consent. Streamlined versions of primary charts will be produced as the process of summarising and coding the data progresses. Key terms and phrases will be retained while repetition and extraneous text will be removed. Overarching themes will be identified within which similarities and differences will be explored.

### Dissemination

The Bristol Young People’s Advisory Group (YPAG) comprise young people aged 10–17 years who are interested in healthcare and research. They meet regularly to help researchers with their projects. Bristol YPAG have been consulted about the design of the study and participant materials. They will also be invited to an event at the end of the study to consider findings and recommendations with the young people, parents, immunisation nurses and school staff involved in the study.

We will produce a report with recommendations in relation to self-consent in conjunction with key stakeholders. This will be presented at events for relevant healthcare practitioners. At least two papers will be written for publication in peer-reviewed journals, and for presentation at academic conferences. We will also summarise the results to share with participating immunisation nurses, school staff, young people and their parents as requested.

## Summary

There is currently a paucity of research into the impact of self-consent for adolescent vaccinations. In relation to the HPV vaccination programme, statistical evidence suggests young women from socially disadvantaged groups are less likely to receive the vaccine, and qualitative evidence suggests that the requirement for written parental consent acts as a barrier. This could lead to increased health inequalities. Written parental consent is not a requirement for many of these young women, although self-consent procedures for younger adolescents are not without controversy. School staff and immunisation nurses appear more comfortable with the vaccine being administered following receipt of a signed parental consent form. An opportunity has arisen in the south-west of England for academic researchers and public health practitioners to work together to examine the process and impact of new self-consent procedures as they are developed and implemented, and to produce recommendations for good practice. Although this research focuses on the HPV vaccination programme, the findings may be relevant to other vaccines that are offered to adolescents in school settings.

## Supplementary Material

Reviewer comments

Author's manuscript
