# Supplementary material for: Impact and acceptability of self-consent procedures for the school-based human papillomavirus vaccine: a mixed-methods study protocol
Source: BMJ Open. 2018 Mar 3;8(3):e021321. doi: 10.1136/bmjopen-2017-021321 (PMC5879539; doi:10.1136/bmjopen-2017-021321)
Supplement: Reviewer comments [file bmjopen-2017-021321.reviewer_comments.pdf]

PEER REVIEW HISTORY

BMJ Open publishes all reviews undertaken for accepted manuscripts. Reviewers are asked to complete a checklist review form (**http://bmjopen.bmj.com/site/about/resources/checklist.pdf**) and are provided with free text boxes to elaborate on their assessment. These free text comments are reproduced below.

ARTICLE DETAILS

**TITLE (PROVISIONAL)**	The impact and acceptability of self-consent procedures for the schools-based human papillomavirus vaccine: a mixed-methods study protocol
**AUTHORS**	Audrey, Suzanne; Batista, Harriet; Ferrie, Jo; Evans, Karen; Bell, Michael; Yates, Julie; Roderick, Marion; Macleod, John; Hickman, M

This article was not externally reviewed at BMJ Open. Protocols that have been independently assessed prior to submission to BMJ Open are usually fast-tracked to publication on the grounds that further substantial changes will not be possible. This independent assessment will usually be external, independent review for both a substantial grant award from a non-commercial or government funder and ethics approval.
